# Supplementary material for: Electrodialytic Desalination of Tobacco Sheet Extract: Membrane Fouling Mechanism and Mitigation Strategies
Source: Membranes (Basel). 2020 Sep 21;10(9):245. doi: 10.3390/membranes10090245 (PMC7559822; doi:10.3390/membranes10090245)
Supplement: Supplementary file 1 [file membranes-10-00245-s001.pdf]

membranes

Supplementary Materials

Supplementary Materials: Electrodialytic Desalination of Tobacco Sheet Extract: Membrane Fouling Mechanism and Mitigation Strategies

Shaolin Ge [1], Zhao Zhang [1], Haiyang Yan [2,3], Muhammad Irfan [2], Yingbo Xu [1], Wei Li [3], Huangying Wang [2] and Yaoming Wang [2,3,*]

1 China Tobacco Anhui Industrial Co., LTD, Hefei 230088, China; slge@mail.ustc.edu.cn (S.G.); zhaoz@mail.ustc.edu.cn (Z.Z.); xuybah@hotmail.com (Y.X.)
2 CAS Key Laboratory of Soft Matter Chemistry, Laboratory of Functional Membranes, School of Chemistry and Materials Science, University of Science and Technology of China, Hefei 230026, China; engr_muhammad.irfan@hotmail.com (M.I.); oceanyan@ustc.edu.cn (H.Y.); why921@mail.ustc.edu.cn (H.W.)
3 Hefei ChemJoy Polymer Materials, Co., LTD, Hefei 230601, China; liwei8991@126.com
* Correspondence: ymwong@ustc.edu.cn (Y.W); Tel.: +86-551-6257-8537

S1. Scanning electron microscopy- energy dispersive spectroscopy (SEM-EDS)

Membrane morphology was characterized by using scanning electron microscopy (XT30ESEM-TM-PHILIP), and the distribution of the elements was analyzed with energy dispersive spectroscopy (EDS).

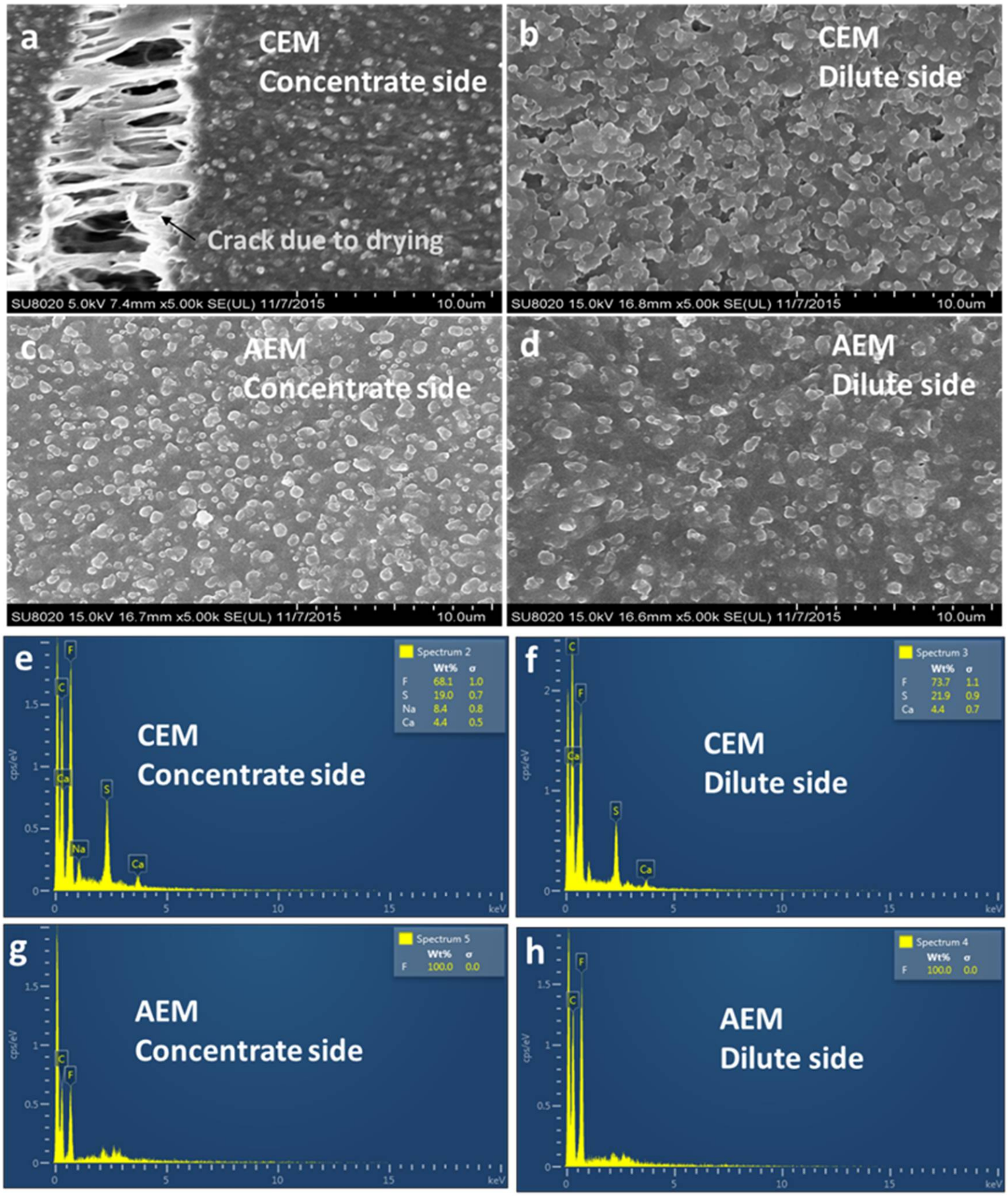

Figure S1. The SEM-EDS images of the fouled membranes: (**a**) and (**e**), CEM, Concentrate side; (**b**) and (**f**), CEM, Dilute side; (**c**) and (**g**), AEM, Concentrate side; (**d**) and (**h**), AEM, Dilute side)
